# Identification and Current Palaeobiological Understanding of “Keratosa”-Type Nonspicular Demosponge Fossils in Carbonates: With a New Example from the Lowermost Triassic, Armenia

**DOI:** 10.3390/life12091348

**Published:** 2022-08-30

**Authors:** Cui Luo, Yu Pei, Sylvain Richoz, Qijian Li, Joachim Reitner

**Affiliations:** 1 State Key Laboratory of Palaeobiology and Stratigraphy, Nanjing Institute of Geology and Palaeontology and Center for Excellence in Life and Paleoenvironment, Chinese Academy of Sciences, Nanjing 210008, China; 2 Department of Geosciences, University of Tübingen, 72076 Tübingen, Germany; 3 Department of Geology, Lund University, Sölvegatan 12, 223 62 Lund, Sweden; 4 Department of Geobiology, Centre of Geosciences, University of Göttingen, Goldschmidtstraße 3, 37073 Göttingen, Germany; 5 Göttingen Academy of Science and Humanities, Theaterstraße 7, 37077 Göttingen, Germany

**Keywords:** Verongimorpha, Keratosa, carbonates, shale, Cambrian, Triassic

## Abstract

Structures similar to fossilized nonspicular demosponges have been reported in carbonates throughout the Phanerozoic and recently in rocks dating back to 890 Ma ago. Interpretation of these records is increasingly influential to our understanding of metazoans in multiple aspects, including their early evolution, the ecology in fossil reefs, and recovery after mass extinction events. Here, we propose six identification criteria of “Keratosa”-type nonspicular demosponge fossils based on the well-established taphonomical models and their biological characteristics. Besides, sponge fossils of this kind from the lowermost Triassic of Chanakhchi (Armenia) are described with a 3-D reconstruction to exemplify the application of these criteria in recognition of such organisms. Subsequently, the state-of-the-art understanding of the taxonomy and evolution of these fossil sponges, a previously poorly addressed topic, is summarized. The morphology of the Triassic Chanakhchi fossils indicates an affinity with verongimorphs, a group that may have evolved by Cambrian Age 3. Other than that, further efforts are encouraged to forge quantitative criteria based on the here proposed descriptive version and to explore the taxonomic diversity and evolutionary details of these fossil nonspicular demosponges.

## 1. Introduction

The fossilized fibrous skeletal frame of nonspicular demosponges was first recognized in carbonates based on Triassic and Devonian examples in a study initially aimed to provide references for the search for Precambrian nonmineralized ancestral animals [1,2]. Since then, similar structures have been extensively reported [3,4]. Following the sponge interpretation, some of these records were thought to have revealed previously unexpected ecological and evolutionary facts about these organisms: they were able to build microbialite-like bioconstructions [5,6,7], bloomed in the aftermath of reef deterioration events in the middle Cambrian and earliest Triassic [8,9,10,11,12,13,14], and may have been present since 890 Ma ago [15], significantly preceding the estimated age of 720 Ma for the emergence of the poriferan lineage (e.g., [16]). However, the sponge interpretation has been questioned, at least in some examples. Proposed alternative interpretations include lithistid sponges [17], Wedl tunnels [18], *Lithocodium* [19], amalgamated micritic clots [20], and metazoan burrows [21].

The discussed nonspicular demosponges possess organic fibrous skeletons and conform to the definition of “Keratosa” in Minchin [22]. Living taxa with such morphological characteristics are now assigned to the subclasses Keratosa and Verongimorpha based on phylogenomic studies [23,24,25]. Although it is difficult to study the exact taxonomy of these organisms in fossil materials due to the lack of histological, biochemical, and genomic information, the family Vauxiidae from Cambrian shale Lagerstätten has been assigned to the subclass Verongimorpha without many controversies because of its exceptionally preserved chitinous cored skeletal fibres [26,27]. By contrast, let alone taxonomy, the recognition and interpretation of these sponge fossils in carbonates are still controversial. Although differences between nonspicular demosponge fossils and superficially similar structures of other origins have been discussed in early literature [2,6], ambiguity remains when analysing specific examples [20].

Since the accurate identification of these nonspicular demosponge fossils in carbonates becomes more and more influential to our understanding of metazoan evolution and paleoecology (as introduced in the beginning), it is necessary to re-examine and refine their recognition criteria. In addition, as data accumulate, the taxonomy, evolution, and other biological features of these organisms need to be addressed.

This study tries to extract a set of recognition criteria of “Keratosa”-type nonspicular demosponge fossils in carbonates based on the established taphonomic models and morphological characteristics of these organisms. Although these criteria are not yet quantitative for some reasons (discussed in Section 3.2), they reflect the most intrinsic morphological characteristics of these sponges and can be used to identify well-preserved examples and exclude many alternative interpretations. In addition, these criteria provide a good stepping-stone to establish more quantified standards. A fossil example from the lowermost Triassic carbonates is analysed to demonstrate the application of these criteria and methods. Finally, the state-of-the-art understanding of the taxonomy and early evolution of these nonspicular demosponge fossils are addressed.

## 2. Materials and Methods

As the basis of proposing identification criteria, the established taphonomic models of these organisms were first revisited. Then the six recognition criteria were proposed based on published data to avoid any circular argument in this article. They are generally a modification of the four morphological characteristics that Luo and Reitner [6] used to identify nonspicular demosponge fossils in thin sections. The information involved in the improvements can all be found elsewhere in the published descriptions in Luo and Reitner [2,6] and Luo [3].

To demonstrate the application of the proposed recognition criteria, a new example of “Keratosa”-type nonspicular demosponge fossil was analysed. The investigated materials were collected from the Chanakhchi (formerly also known as Zangakatun or Sovetashen) section in SW Armenia [9,28]. The section was located on the western margin of the Cimmerian microcontinent between the Neotethys and Palaeotethys oceans during the Early Triassic [29]. Carbonates in this section were deposited between fair weather and storm wave base on a distal and low-relief open-marine ramp [9,11]. Two units of sponge-microbial bioherms are present above the strata that represent the end-Permian mass extinction records. The lower one is 5 m thick, expanding from the postextinction Permian to the second conodont zone of the Griesbachian, Induan (*Isarcicella isarcica* zone). The second one, late Griesbachian in age, is 13 m thick and encloses several thrombolites and dendrolitic biostromes and bioherms. The thickest microbialite in the Chanakhchi section is from the upper microbialitic interval and is up to 8 m wide and 12 m thick [9]. Massive asymmetric thrombolitic domes are distinct in the lower half of the bioherm, followed by several thrombolitic biostromes and again several thrombolitic and dendrolitic mounds. The sample studied here comes from the lowest of the upper dendrolitic part and corresponds to Sponge Facies 3 and sample 81 described in Friesenbichler et al. [9].

Thin sections were examined using a Zeiss SteREO Discovery.V8 microscope and photographed using the attached AxioCam MRc 5-megapixel camera. A chosen piece of the samples was cut into an approximately 30 × 30 × 5 mm chip for grinding tomographic analyses. It was mounted on a glass and then serially ground using the same method as that described in Luo and Reitner [2]. One-hundred polished planes were photographed using the same set of microscope and camera system mentioned above. A Mitutoyo micrometre was used to control interplane distances. The average distance was 9.6 ± 1.1 μm (Supplementary File S1A). Although the 36th and 37th images were recorded the same by mistake, this does not much affect the outcome.

The obtained images were aligned using Adobe Photoshop and converted to the grey-scale mode (Supplementary File S1B). A 3.09 × 2.33 mm area, which includes a well-preserved skeletal frame and aquiferous canals, was cropped for further processing (Supplementary File S1C). The brightness and contrast of these images were adjusted one by one using GIMP 2.10.14. The dark areas in the background, which would otherwise affect the visualization of the skeletal frame, were meanwhile masked by a light grey colour (Supplementary File S1D). The resulting stack was visualized using Voreen 5.2.0 (voreen.uni-muenster.de) (Supplementary Files S1D and S3A).

Two smaller areas within the 3.09 × 2.33 mm area were processed similarly, but with the background more carefully removed in GIMP to better visualize the aquiferous canals and skeletal meshes (Supplementary File S1E–H).

All fossils and thin sections illustrated in this study are deposited in the Geoscience Museum of the University of Göttingen. Electronic data generated in the grinding and image processing are available in Supplementary File S1.

## 3. Results

### 3.1. Preservation of “Keratosa”-Type Demosponges in Carbonates

**Figure 1:**
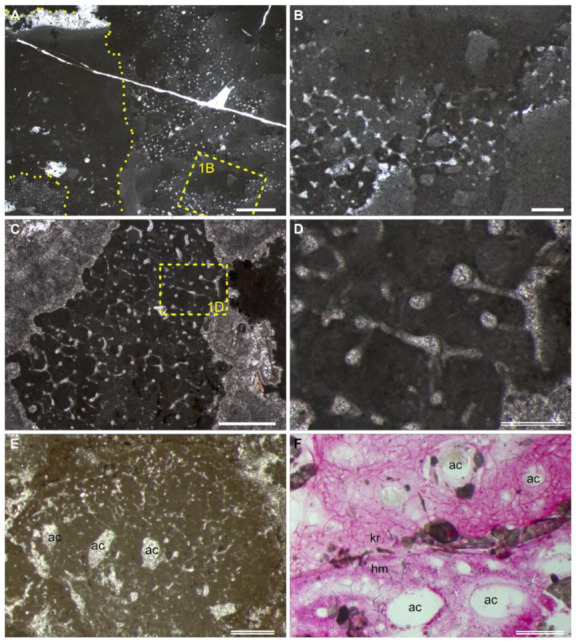
Preservation of sponges in carbonates. (**A**–**D**) A hexactinellid (**A**,**B**) and a lithistid (**C**,**D**) fossil from an Albian (Lower Cretaceous) mud mound in Araya, Spain [3,35]. Rectangles in (**A**) and (**C**) are enlarged in (**B**) and (**D**), respectively. The region enclosed in the dashed line of (**A**) on the left part of the image is a spongocoel filled with geopetal deposits. (**E**) A nonspicular demosponge fossil from the Carboniferous Clifton Down Limestone, UK [6]. (**F**) Histological thin section of a living nonspicular demosponge (kr) and a homoscleromorph (hm) from the Lizard Island, stained in basic fuchsine. Comparable aquiferous canals (ac) are indicated in (**E**,**F**). Thin section number: (**A**–**D**), AR; (**E**), BH10; (**F**), Liz234. Scale bars: single line = 2 mm; double line = 0.5 mm.

Similar to the preservation of siliceous sponges (Figure 1A–D), the fibrous skeleton of “Keratosa”-type nonspicular demosponges is often moulded in a micritic matrix and replaced by microspars (Figure 1E). The organic skeletons of nonspicular demosponges are composed of spongin and/or chitin that are more resistant to biodegradation than other soft tissues [30,31,32,33]. This allows them to be moulded in syndepositional micrites and then replaced by calcite spars, the same as the taphonomic processes that siliceous spicules are subjected to [34].

The syndepositional micrites could be accumulated through two different paths. The first is the precipitation of automicrites during the decay of the sponge soft tissue. These processes and the resulting fossils have been repeatedly observed in modern and palaeontological examples [36,37,38,39]. The main trigger of the rapid automicrite formation has been attributed to the raised alkalinity and the presence of a nucleation template due to the decay of sponge tissues in restricted microenvironments [37,40,41]. Some studies emphasize the role of organic sorbent in carbonate nucleation [39,42]. Generally, high alkalinity in the seawater is favourable for the automicrite precipitation.

The second path is the deposition of allomicrites. Take the hexactinellid fossil in Figure 1A,B as an example. The skeletal frame seems to be first moulded by automicrites, which show a patched texture. Then the spongocoel was filled by allomicrites, which form geopetal structures. The organic skeletons of nonspicular demosponges are for a longer time resistant to degradation and could be washed out from other soft tissues of the dead sponge (imagine a piece of natural bath sponge). Rapid burial of these skeletons with fine sediments would be favourable for their preservation.

### 3.2. Recognition Criteria of “Keratosa”-Type Demosponges in Carbonates

A perfect validation for the presence of these sponges would be an iconic sponge body with a spongocoel surrounded by an anastomosing fibrous skeletal frame. However, regardless of the fact that many shallow water and cave-dwelling nonspicular demosponges are encrusting and formless [43,44], a sponge body erected in the seawater is difficult to be completely fossilized according to the introduced taphonomic models.

For those fossils formed following the first taphonomic path, Luo and Reitner [6] used four morphological characteristics to identify them in thin sections and exclude other interpretations. These characteristics are here advocated again with a few refinements based on the information already provided in Luo and Reitner [2,6].

**Figure 2:**
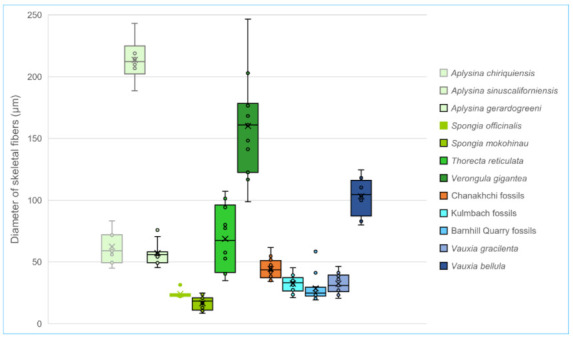
Thicknesses of skeletal fibres of a few living and fossil nonspicular demosponges compared with the Chanakhchi fossils. The greenish boxes represent living taxa, the orange box represents the Chanakhchi fossils, and the blueish boxes represent other fossil examples. Among them, *Aplysina* and *Verongula* belong to the order Verongiida, subclass Verongimorpha; *Spongia* and *Thorecta* belong to the order Dictyoceratida, subclass Keratosa. For original measurements and data sources, see Supplementary File S2.

- Fibrous skeletons are preserved as microspar-cemented moulds in homogeneous automicrites.
- The skeletal fibres form an anastomosing network extending three-dimensionally in the micritic aggregation with a generally uniform density.
- The skeleton persists a uniform thickness along each fibre. In the whole skeletal frame, the fibre thicknesses either change gradually or exhibit regular orders or hierarchies. For reference, the diameters of skeletal fibres in living nonspicular demosponges vary from a few to hundreds of micrometres, with many being around tens of micrometres thick (Figure 2; Supplementary File S2).
- The fibrous network is constrained in the micritic aggregation and exhibits fibres lining the border of the aggregation, such as between the sponge body and the hard substrates and wrapped particles.
- There are no desmas (cf. Figure 1D), incorporated spicules, or orthogonal symmetry (Figure 1A,B) in the fibrous network that could indicate an affinity of spicular sponges.
- Water canals of the sponge aquiferous system are sometimes preserved (Figure 1E,F; more discussion in Section 4.1). If present, they add credits to the sponge interpretation.

The above characteristics II–III restrict the recognition of nonspicular demosponges to those groups that possess regular anastomosing skeletons. Although many living taxa have dendritic skeletal frames and irregularly knotted fibres (e.g., family Pseudoceratinidae) [45], they have never been invoked to interpret fossil structures. Therefore, these forms are not discussed here.

The descriptive “uniform density” of the skeletal network (characteristic II) and “uniform thickness” of fibres (characteristic III) have the potential to be specified with a quantitative expression. However, that requires a thorough census of the morphological variation in living taxa, which this study was unable to accomplish due to the huge workload of literature digging and the lack of accessibility to necessary collections.

For sponge skeletons preserved following the second path, many of these characteristics are either inapplicable or difficult to recognize, including automicrites, the margin of the fibrous network, and aquiferous canals. The morphology of the 3-D fibrous network is almost the only thing that can be counted on. Preservation like this, as well as diagenetic alterations, could wipe away important biological information and introduce uncertainties to the identification of nonspicular demosponge fossils.

### 3.3. Observation of the Chanakhchi Fossils

**Figure 3:**
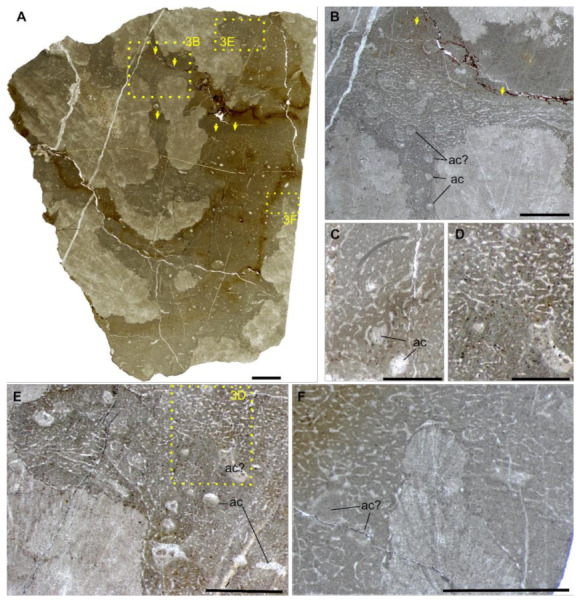
The sponge fossils from Chanakhchi (Armenia). (**A**) Overview of the sponge fossils encrusting over dendrolites. Areas in rectangles are enlarged in (**B**,**E**,**F**), respectively. Yellow arrows in (**A**,**B**) indicate the upper surface of the organisms. (**B**–**F**) Closer view of the skeletal fibres, aquiferous canals (ac), and possibly previous aquiferous canals fully filled by a later generation of deposits or sponge tissues (ac?). (**D**) is enlarged from the yellow rectangle of (**E**). Thin section number: (**A**,**B**,**D**–**F**), GZG-number INV.143; (**C**), GZG-number INV.142. Scale bars in (**A**) = 5 mm, in (**C**,**D**) = 1 mm, in other images = 2 mm.

The thin sections contain bushy and rounded mesoclots, which are composed of microbially induced crystal aggregates [9]. They grow on each other to form dendrolitic columns. The structures interpreted as sponge fossils (Section 4.2) grow in the interspace of these thrombolites and dendrolites, sometimes with their upper surface preserved (Figure 3A,B). No other microfossils have been observed in these materials.

**Figure 4:**
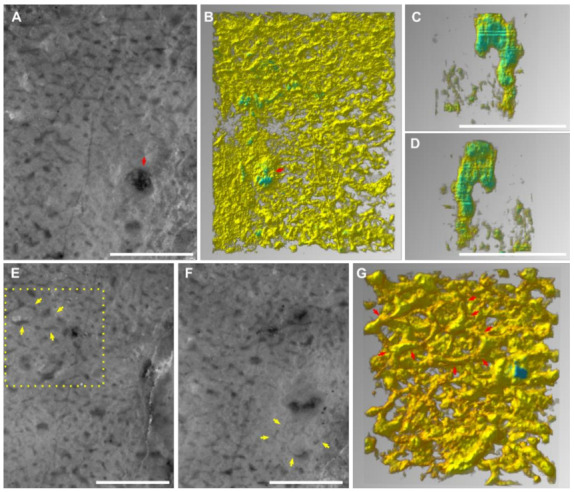
Serial grinding and 3-D reconstruction of a 3.09 × 2.33 × 0.96 mm volume of the Chanakhchi sponge fossil. (**A**,**B**) A serial grinding photo (**A**) and the corresponding 3-D reconstruction (**B**) show the presence of aquiferous canals (red arrows). The images of (**A**,**B**) mirror each other because (**A**) was the last plane of the serial grinding, and (**B**) shows the sole of the reconstructed block. (**C**,**D**) Lateral views of the isolated 3-D structure of the aquiferous canal, with the last serial grinding plane on the top. (**E**,**F**) Serial grinding photos showing the existence of regular hexagonal meshes (yellow arrows). (**G**) A 3-D reconstruction of the region in the yellow rectangle in (**E**), showing the 3-D structure of the hexagonal meshes (red arrows). All scale bars = 1 mm.

The spar-cemented fibrous networks of these fossils are embedded in homogeneous grey micrites (characteristic I). The fibres exhibit consistent thickness on each string, with diameters in the range of 37–51 μm (characteristic III; Figure 2; Supplementary File S2). They can line up the boundary between the organism and alien objects, such as the mesoclots and wrapped particles (characteristic IV; Figure 3B,C,E,F). Neither spicules nor desmas have been observed in thin sections (characteristic V). Although the fibre thicknesses vary in a range, there was no clear separation of fibre hierarchies like that in many living dictyoceratid skeletons [46]. Serial grinding and 3-D reconstruction confirmed that these microspar-cemented fibres were indeed part of a three-dimensional network (characteristic II; Figure 4; Supplementary File S3). Most meshes in this network appear to be randomly polygonal, although some of them are more regularly hexagonal (Figure 4E–G).

Many irregular but mostly tubular cavities are scattered in these fossils. They are one magnitude thicker than the skeletal fibres (200–300 μm wide in thin sections with extreme widths of over 500 μm, Supplementary File S2), cemented by calcite spars and sometimes containing geopetal fillings (Figure 3B–F). These structures are different from fenestral fabrics in showing a rounded outline in cross sections and lacking a layered distribution pattern. By 3-D reconstruction, these structures are proved blind-ending tubes, resembling the architecture of aquiferous canals in living sponges [47] (characteristic VI; Figure 4C,D). There are also rounded patches of the same scale as these cavities, which are, however, filled with skeletal fibres and paler micrites (Figure 3B,F). These may represent either old aquiferous canals filled by a later generation of sponge tissues or different generations of micrite deposition during early diagenesis, similar to the paler patches in the spicular sponge fossils in Figure 1B,C.

## 4. Discussion

### 4.1. Differentiating “Keratosa”-Type Demosponge Fossils from Other Similar Structures Based on Proposed Criteria

As introduced in the beginning, several sorts of structures are easily confused with “Keratosa”-type demosponge fossils. Due to varied preservation types and quality, there is indeed a spectrum of morphological intermediateness between these demosponge fossils and structures of other origins. This holds true for nearly all sorts of fossil materials, and palaeontologists often cope with this problem by analysing only the best specimens. For this reason, when discussing the differentiation between “Keratosa”-type demosponge fossils and similar structures, we refer to the best specimens that exhibit all the proposed characteristics I–VI. Such fossil materials are not just a conception or fantasy. The Chanakhchi fossils have shown an example of them (Section 4.2).

The morphological differences between the fibrous sponge skeletons and fossilized cyanobacteria and fungal hyphae have been discussed in Luo and Reitner [2,6]. Those arguments are still valid today. Fossilized filamentous cyanobacteria often have a dark lining along the fibres, representing their durable sheaths [48]. In contrast, the moulds of sponge skeletal fibres do not have such a lining (characteristic I). The recognition of fossilized fungal hyphae networks is normally based on the presence of septa [49] and/or various reproductive structures, such as conidia or chlamydospores [50,51], which were not seen within the sponge skeletal fibres.

Morphological characteristics II–IV reflect the biological features of a sponge skeleton. The skeletal frame is a supportive structure and thus extends three-dimensionally with specific patterns of mesh size and shape and fibre thicknesses (characteristics II–III). Although the patterns vary among different taxa, the fibrous network must have a clear border against the surroundings (characteristic IV), for it belongs to an individual animal. In contrast, Wedl tunnels, the labyrinth-like micrometre-scale canals, were bored by fungi or cyanobacteria to seek nutrients [52]. They do not have the geometric patterns and the self-constraining borders as the sponge skeletons. *Lithocodium* is a problematicum possessing septate and branching filaments [53,54]. These filaments are not network forming, and their diameters decrease with successive branching.

Compared with these nonporiferan structures, the skeleton of other spicular sponges could be more easily confused with that of nonspicular demosponges [17]. In this case, characteristic V could be consulted to discriminate these different skeletons. The siliceous skeletal frame of lithistid sponges is composed of articulated desmas [55]. These hypersilicified spicules have complex terminal expansions (zygoses) and are recognizable in the carbonate fossils (Figure 1D) [56]. Fused skeletons of hexactinellids show orthogonal symmetry in the grids (Figure 1B). In both groups, the fused siliceous skeleton can be, but is not always, associated with unfused spicules. Moreover, some haplosclerid demosponges (e.g., Family Callyspongiidae) possess anastomosing organic skeletons similar to that of dictyoceratids [57]. Nevertheless, the skeletons of the former are often cored with monaxon spicules and/or foreign materials.

It has been addressed previously that fenestral structures, compacted peloids, and amalgamated micritic clots do not form cavities with the regularity or architectural pattern seen in sponge skeletons (characteristics II–IV) [2,6]. The Silurian structures described in Kershaw et al. [20] are in some parts similar to nonspicular demosponge fossils (e.g., Figure 3, 4 in [20]) while also showing irregularities in other parts of those materials (e.g., Figure 2, 5 in [20]). For the preservation problem stated at the beginning of this section, the interpretation of these structures is pending. Three-dimensional reconstruction and quantitative criteria, which are both lacking at this moment (Section 3.2), are critical for evaluating whether these structures fall into the scope of sponge skeletons. On the other hand, experiments or observation of modern examples is needed to prove whether compacted micritic clots do form uniform and complex cavity networks conforming to characteristics II–VI.

The Cambrian microburrows mentioned in Kris and McMenamin [21] were initially identified following an earlier description of similar structures in Wood et al. [58] [59]. However, the diameter of these microburrows is 100–500 μm, much thicker than the fibre thicknesses of so far recognized fossil nonspicular demosponges [2,6,13] (Figure 2). Microburrows from the Cambrian of Inner Mongolia [60] and the Ediacaran–Cambrian transition of Brazil [61] can be as small as tens of micrometres in diameter. However, 3-D reconstructed examples show that they do not form the 3-D network like the sponge skeletons (characteristic II) [61]. The graphoglyptid trace fossils referred to in Kris and McMenamin [21], particularly *Palaeodictyon*, typically occur in deep-water siliciclastic sediments. They are composed of tunnels with millimetric to centimetric diameters. The tunnels form regular hexagonal meshes mainly on two-dimensional surfaces parallel to the sea bed [62,63,64].

Despite the examples above, skeletons of nonspicular demosponges can still appear similar to many other structures in thin sections, such as plant roots moulded in caliche nodules (pages 728–729 in [65]), the trabecular meshwork in echinoderm skeletal plates [66], and some scleractinian corals in certain cross sections [67]. Therefore, reliable recognition of nonspicular demosponges cannot be merely based on thin sections or thin section photos. All available information from outcrops to microfacies analyses must be considered synthetically to minimize ambiguity.

The above comparison between nonspicular demosponge fossils and alternative interpretations are mainly based on skeletal morphology (characteristics I–V). The presence of the aquiferous system (characteristic VI) increases the credibility of the sponge interpretation. Choanocyte chambers and ramifying canals conduct fluids through the sponge body and are more essential characteristics of sponges than the skeleton [68]. The canals are possible to be preserved in carbonates following the first taphonomic path described in Section 3.1 (e.g., [36,69]). In this model, micrites tend to first nucleate on the organic template released by the decay of the soft tissue [37], leaving the water canals vacuous. These empty spaces could be subjected to later generations of deposits. The most often and readily recognized example of the aquiferous system in sponge fossils is the spongocoel (Figure 1A), while other parts of the aquiferous canals vary a lot in size (from tens of micrometres to macroscopic) and organization (e.g., [47,68]) and may be misinterpreted as other structures.

### 4.2. Identifying Chanakhchi Fossils as Nonspicular Demosponges

According to the description in Section 3.3, the morphology of the Chanakhchi fossils clearly fits the identification criteria I–VI proposed in Section 3.2, and none of the alternative interpretations discussed in Section 4.1 could explain the combination of all these characteristics.

The development of the crystal aggregates, on which the sponge fossils encrust (Section 3.3), indicates a carbonate-oversaturated setting [9]. Therefore, the presence of the Chanakhchi sponge fossils fits the taphonomic model of “Keratosa”-type nonspicular demosponges in carbonates—micrites could be rapidly precipitated from the oversaturated seawater and/or porewater once the decay of the sponge tissue releases organic substrates for nucleation. Moreover, the preservation of the Chanakhchi material is comparable with that of the hexactinellid fossil illustrated in Figure 1A,B. In both fossils, the skeletal frame was first moulded by automicrites, and then the large aquiferous canals were filled by allomicrites.

### 4.3. “Keratosa”-Type Demosponge in the Fossil Record: Known and Unknown

Although occurrence data of “Keratosa”-type sponge fossils or similar structures are accumulating [4], not all of them were illustrated with enough information or proper preservation as required to evaluate using criteria I–VI (e.g., [18,70]). However, examples conforming criteria I–V are indeed known throughout the whole Phanerozoic (e.g., Cambrian [5]; Ordovician [71]; Devonian [2]; Carboniferous [6]; Triassic, this study; Miocene [72]). In some of these examples ([5,6] and this study), characteristic VI is also present.

The recently reported alike structures from the 890-Ma-old Little Dal Group [15] look nearly indistinguishable from the middle Cambrian to Ordovician analogues in terms of characteristics I–V in thin sections. This raises a dilemma. On the one hand, there are yet no competitive alternative interpretations for these fossils other than nonspicular demosponges. On the other hand, if this interpretation is true, it is hard to imagine how these organisms kept their shape nearly unchanged through around 400 million years.

Regardless of this oldest example, perhaps the early Cambrian fossil record is the best starting point to explore the evolutionary history of these animals. After all, the most widely acknowledged fossil representatives of these demosponges are vauxiids from the Konservat-Lagerstätten of early to middle Cambrian shales [26,73,74,75]. The morphological characters of sponge skeletal frames can easily be obtained in this preservation art compared with that in carbonates. Vauxiids were assigned to the order Verongiida, subclass Verongimorpha, because their skeletons seem to be constructed with cored fibres composed of chitin [26,27], consistent with the skeletal characters of living verongiids [34,76,77]. Nevertheless, the vauxiids from Cambrian Ages 3 and 5 of South China are all associated with silicification in the fibrous skeletons [73,74,75]. It requires further efforts to determine whether the silicification is of biological or diagenetic origin and whether this will affect the original phylogenetic assignment.

**Figure 5:**
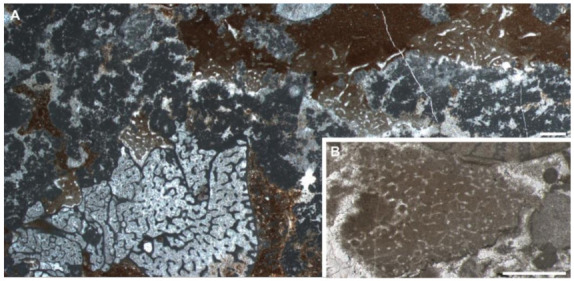
*Dictyocyathus translucidus* from the Tommotian archaeocyath-calcimicrobe reefs of Siberia (cf. [79]). Thin section number: (**A**), CHD; (**B**), BB3. All scale bars = 1 mm.

In records prior to Cambrian Age 2, some fossils from the Tommotian carbonates of Siberia were once interpreted as “Keratosa-”-type nonspicular demosponges (Figure 5) [3]. These fossils were later diagnosed as the archaeocyath *Dictyocyathus translucidus* [78], a unique archaeocyath species whose skeleton is always preserved as moulds filled with sparitic cements [79,80,81]. This exceptionality in archaeocyaths was previously attributed to originally aragonitic mineralogy in the skeleton [79,80]. However, this interpretation does not explain the source of the micrites that often mould the spar-cemented skeletons. Illustrations of *D. translucidus* are very sparse in published studies. At least in the observed example in Figure 5A, *D. translucidus* is preserved next to other archaeocyaths and *Epiphyton* shrubs that are devoid of micrite fillings in the inner- and interspaces. In this case, the nonspicular demosponge interpretation of *D. translucidus* seems to be reasonable from a taphonomic point of view.

Moreover, a recent study of fossils from the Guanshan Biota (Cambrian Age 4) suggested that archaeocyaths of the suborder Archaeocyathina, to which *D. translucidus* also belongs, are morphologically very close to vauxiid sponges [81]. The investigated Guanshan fossils can be comfortably assigned to either of these groups. The only problem is still the skeletal composition: the fibrous skeletons in the Guanshan fossils are silicified, neither carbonate as that of archaeocyaths nor organic as iconic vauxiids. It is suggested that archaeocyaths are probably a polyphyletic rather than a monophyletic group.

Noticeably, the skeletons of vauxiids and archaeocyathine archaeocyaths all show repeatedly occurring hexagonal-dominant meshes [81]. In living nonspicular demosponges, taxa that typically exhibit hexagonal meshes are verongimorphs, such as *Aplysina* [82] and *Verongula* [83]. Although hexagonal meshes can also be sporadically present in the skeletal frame of some dictyoceratids, such as *Spongia officinalis* [83], the Cambrian fossils do not show the hierarchical separation of fibres that is common in dictyoceratids [25,46,83]. Thus, these skeletal characters with nonhierarchical fibres and hexagonal meshes support the previous assignment of vauxiids to the order Verongiida. Following this comparison, the Chanakhchi sponges may also belong to Verongimorpha because their skeletons are anastomosing and without the separation of fibre hierarchies, although the frequency and regularity of hexagonal meshes in these fossils are incomparable with that of vauxiids.

Since the subclasses Verongimorpha and Keratosa are sister groups, the order Dictyoceratida and part of order Dendroceratida are also expected to be present among the fossil specimens with reticulate fibrous skeletons. More morphological studies are required to explore further taxonomic and evolutionary details in these fossil materials.

## 5. Conclusions

By proposing the six identification criteria, commenting on alternative interpretations, and analysing a representative fossil example, this study underpins the point that “Keratosa”-type nonspicular demosponges were indeed preserved in Phanerozoic carbonates. Recognizing these fossils requires a synthetical consideration of information from outcrops to lab analyses and from taphonomy to ecology so that ambiguity and confusion can be minimized. Quantitative criteria will help to reduce ambiguity in the identification of nonspicular demosponge fossils, and the proposed descriptive criteria could be a stepping-stone for achieving this goal.

Although nonspicular demosponge fossils are known throughout the Phanerozoic and possibly since the Neoproterozoic, their taxonomy and evolutionary pattern are still poorly understood. However, if verongimorphs had evolved by the Cambrian Age 3, as discussed, some taxonomic diversity is expected to be present in the fossil record of nonspicular demosponges. Comprehensive morphological studies combined with modern spongiology are required to reveal more details on this issue in the future.

## Data Availability

The data presented in this study are openly available in Figshare at https://doi.org/10.6084/m9.figshare.c.6089724.v3, accessed on 26 August 2022.

## Supplementary Materials

The following are available online at https://doi.org/10.6084/m9.figshare.c.6089724.v3, accessed on 26 August 2022 , Supplementary File S1: Raw data of the 3-D reconstruction. (A) Grinding records. (B) Originally photographed images aligned by Photoshop. (C) Cropped and grey-scale-adjusted images used for the reconstruction of Supplementary File S3A. (D) Files generated during the processing of Supplementary File S1C using GIMP and Voreen. (E) Cropped and grey-scale-adjusted images used for the reconstruction of Supplementary File S3B. (F) Files generated during the processing of Supplementary File S1E using GIMP and Voreen. (G) Cropped and grey-scale-adjusted images used for the reconstruction of Supplementary File S3C. (H) Files generated during the processing of Supplementary File S1G using GIMP and Voreen. Supplementary File S2: (A) Measurements of the fibre thickness in some living and fossil nonspicular demosponges. (B) Images that were measured. Supplementary File S3: (A) 3-D view of the region illustrated in Figure 4A,B,E,F. (B) 3-D view of the aquiferous canal illustrated in Figure 4C,D. (C) 3-D view of the skeletal frame illustrated in Figure 4G.
